# “Felony assault should stick:” Assaulted EMS responders’ frustration and dissatisfaction with the legal system

**DOI:** 10.1002/ajim.23036

**Published:** 2019-08-16

**Authors:** Jasmine Y. Wright, Andrea L. Davis, Sherry Brandt‐Rauf, Jennifer A. Taylor

**Affiliations:** ^1^ Department of Environmental & Occupational Health Dornsife School of Public Health at Drexel University Philadelphia Pennsylvania

**Keywords:** assault, emergency medical services, felony, firefighter, workplace violence

## Abstract

**Introduction:**

The prevalence of violence to first responders is reported in ranges of approximately 40% to 90%. Pennsylvania has a felonious assault statute to address such violence, but the prosecutorial process has been noted to cause first‐responder dissatisfaction.

**Methods:**

An exploratory qualitative study using individual interviews with snowball sampling was conducted with the Philadelphia District Attorney's office to understand the prosecutorial process when a first responder is assaulted and injured in a line of duty. The Philadelphia Fire Department provided a list of first responders who sustained a work‐related injury from a patient or bystander assault so that particular cases could be discussed during the interviews.

**Results:**

Emergent themes fell into two categories: factors that lead to a charge (prosecutorial merit, intent, and victim investment), and the judge's discretion in sentencing (“part of the job” mentality, concern for the defendant, and the justice system's offender focus). Immediately actionable tertiary prevention recommendations for fire departments, labor unions, and district attorney's offices were developed.

**Conclusion:**

Violence against fire‐based emergency medical service (EMS) responders is a persistent and preventable workplace hazard. While felonious assault statutes express society's value that it is unacceptable to harm a first responder, this study found that such statutes failed to provide satisfaction to victims and that support when going through the court process is lacking. Assaulted EMS responders, their employers, and labor unions would benefit from the recommendations provided herein to help them extract a stronger sense of procedural justice from the legal process.

## 1 INTRODUCTION

### 1.1 Violence experienced by first responders

Emergency medical services (EMS) are an integral part of the United States health care system, yet unique in that services are provided in mobile and field‐based environments as opposed to bricks and mortar. In addition to medical emergencies, EMS providers respond to motor vehicle crashes, fires, crime scenes, and natural disasters. Compounding their routine job demands, EMS providers also face danger in the line of duty from physical assaults by patients and bystanders.^1^, ^2^, ^3^ In addition to the physical injuries sustained from violent acts, the resulting increased mental stress also leads to lower work productivity and decreased job satisfaction.^4^

In a systematic review of the literature, Maguire et al^3^ found a 60% to 80% prevalence of workplace violence among EMS responders. His earlier work found excess mortality^5^ and morbidity^6^ among US EMS workers. The former study found that 57% of the injury cases resulted in lost workdays, impacting workforce productivity. Physical assaults by patients caused about 37% of injuries and fatalities.^2^ Another systematic literature review, published in 2018 by the US Fire Administration, reviewed both the academic and industrial literature to explore how occupational violence to firefighters and EMS responders could be mitigated.^7^ In studies that measured prevalence of violence throughout a first responder's career, between 57% to 93% of EMS responders reported they had experienced either verbal or physical violence at least once in their career.^7^

About 40% of EMS in the United States is provided by fire departments.^8^ In a study with the Philadelphia Fire Department, paramedics had 14‐fold higher odds of assault‐related injury compared to firefighters.^1^ Interviews and focus groups conducted with the injured to better understand the assault phenomenon revealed issues with dispatch, training, community expectations, and the legal system.^1^ The last theme was very compelling in that many first responders felt that those who assault them do not face true punishment for their actions:

And I went to court, and this is where it's disheartening, because that's supposed to be felony assault…And I'm wasting my time going to court two and three times…I knew there was no confidence in the system…I mean, you shouldn't be able to do that to someone who's trying to help you. Felony assault should stick. (John, Firefighter‐EMT)^1^

I know we have a law here that if you assault a paramedic, firefighter, police…you're supposed to be charged with a felony. That rarely ever happens. You know, most of the time, you go in there alone…Our medics go alone…You sit there alone…And [the assailant] will explain how sorry they are and what they did to you. And the judge says, “okay”…And that's all you get. (Jane, Paramedic)^1^

### 1.2 First‐responder felonious assault law

The purpose of this study was to learn about the processing and disposition of cases that come before the Philadelphia, PA District Attotney's (DA's) office when a Philadelphia Fire Department firefighter, emergency medical technician (EMT), or paramedic has been assaulted by a patient or bystander. The Philadelphia Fire Department (PFD) is a 2700‐member department with 63 stations, 55 ambulances, and one of the highest EMS call volumes in the nation. About 71% of the 378 849 annual calls are for EMS services.^9^ It is the largest fire department in Pennsylvania, and one of 16 US (<1%) departments that serve populations over 1 million.^10^ PFD is not typical of most fire departments in the US, as approximately 75% are volunteer (unpaid) and 70% of departments have only one station.^11^

In Pennsylvania, Title 18, Chapter 27, Section 2702 of Pennsylvania Constitution Statute explicitly states that it is a felony to assault a member of the protected class:

A person is guilty of aggravated assault if he: attempts to cause or intentionally, knowingly or recklessly causes serious bodily injury to any of the officers, agents, employees or other persons defined in subsection (c), as a member of the protected class while they are in the performance of duty.^12^

Subsection (c) defines those members in the “protected class” as follows:

Firefighter, probation/parole Officer, the Attorney General, the Governor, District Attorney, Lawyers, Parking Enforcement Officer, any person employed to assist any Federal, State or local law enforcement official, any teaching staff member or school board member, and all emergency medical services personnel. The term “emergency medical services personnel” includes, but is not limited to, doctors, registered nurses, licensed practical nurses, nurse aides, paramedics, EMTs and members of a hospital security force while working within the scope of their employment.^12^

Simple assault is the intentional use of force or violence against another person that involves minor injury or a limited threat of violence. It is normally charged as a misdemeanor. Aggravated assault is a stronger form of simple assault, usually involving a deadly weapon and/or intent to cause serious bodily harm. It is normally charged as a felony. Simple assault against a member of the protected class is treated differently and considered a more serious crime. What would typically be a simple assault is escalated to an aggravated assault and is, therefore, a felony. Theoretically, prosecution under this law would result in either a first‐ or second‐degree charge. However, many first responders feel that those who assault them do not face appropriate punishment for their actions:

Well, the prosecution phase is a sore spot… The resolution to it was very disappointing. The person was able to plead and get some kind of a deal where they didn't serve any jail time. They didn't get put on probation. They were given what they call ARD. I don't think they received a just punishment for what they did. (Unpublished data, Dr. Jennifer Taylor, personal communication)

Through an exploratory qualitative research study with those who prosecute such cases on behalf of the DA office, we sought to understand this prosecutorial pathway in light of the fact that the process led to first‐responder dissatisfaction.

## 2 METHODS

The research team contacted a senior member of the Philadelphia DA's office. After an initial interview, snowball sampling was used to ask for other potential colleagues who had important perspectives relevant to the study goal. We were particularly interested in DAs who had prosecuted an EMS responder case based on a list of assaulted members provided by the fire department. We also sought DAs of various ranks having diverse experiences in trial, charging, and diversion divisions to understand the prosecutorial process. From that initial interview, four additional district attorneys were identified and interviewed. Participants were contacted via phone and email and were invited to participate in the study. Written informed consent was obtained from each participant before the start of each interview. All interviews lasted no longer than 60 minutes. A semistructured interview guide was developed to assist with the collection of the data (Table 1). Questions addressed the job demands and pressures experienced by District Attorneys, how they prosecute cases of first‐responder assault, and their experiences with perpetrators of these assaults. The Drexel University Institutional Review Board approved the study protocol. With the assistance of the Philadelphia Fire Department, a list of first responders who sustained a work‐related injury from a patient or bystander assault was provided to the DA's office so that they could retrieve records and discuss particular cases during their interviews.

**Table 1:** Interview guide for District Attorney's (DA's) staff

Tell me what it is like to work in the office of the DA.
Describe for me the demands of your job, in terms of getting through your caseload.
Do you feel pressure from the public to prosecute these offenders because they are first responders?
Do you think your stress levels at work effect the dispositions given to offenders?
Tell me about the role that the Philadelphia DA's office plays in assaults to first responders.
○ How are cases referred to your office?
What options are at your disposal in the prosecution of these cases?
How much discretion do you have in determining the sentence?
○ Are individuals usually offered Accelerated Rehabilitative Disposition, referred to a mental health facility or social service?
○ Are people that are diagnosed with mental illnesses given a different process in these court cases?
○ What about the homeless population?
Can you explain to me what the Accelerated Rehabilitative Disposition Program is and what purpose it serves?
○ How do you feel when you see these cases being presented?
Tell me what you think is going on with the individuals who make these assaults.
○ Do you think the stress levels of the community contribute to this issue?
Tell me about cases that made it to court and how the charges were decided.
What characteristics of the perpetrator do you think affected the sentence?
○ Mental health status? First‐time offender? Gender, race, neighborhood, etc?
○ How do you think the minority population gets handled when dealing with these cases?
Do you think the law, as currently written, is the best way to handle this issue?
○ If no, what changes do you think should be made to the law in Pennsylvania?
○ Is there an alternative? Are other cities/states doing things differently?

The individual interviews took place in various locations (eg the research team's office or the District Attorney's office). Before this study, the researchers knew none of the participants. The interviews were conducted by all authors, each of whom has training in qualitative methods. Interviews were audio recorded and then transcribed by a professional transcription agency. Once transcribed, all unique identifiers were removed from the transcripts.

A structure was developed by the research team (Table 2) to facilitate the coding of the data using NVivo qualitative data analysis software (version 10, 2012; QSR International Pty Ltd). The authors read each transcript in their entirety and used an iterative process to develop the coding structure. As the transcripts were coded and analyzed, the authors looked for similarities and disparities in points of view. Both JYW and JAT coded all five transcripts using NVivo software and had a 96.7% interrater agreement across all nodes and all sources. Using an iterative approach, the authors reviewed the data and summarized key findings through a series of meetings. JAT and ALD reached consensus on the cross‐cutting themes and patterns within the data.

**Table 2:** Qualitative coding structure

**Individual work perception**: The way District Attorney's perceive their work (stressful, satisfying, busy, and rewarding).
**Job pressures and demands**: Any pressures they experience from the public, law enforcement, or upper level management to prosecute cases.
**Policies**: Policies that are currently put in place for the prosecution of cases.
**Formal process**: The pathway/steps that occur from arrest until the final disposition of a case.
**ARD**: Anything associated with the Accelerated Rehabilitative Disposition Program.
**Informal process**: The steps that include: determining intent of crime, gathering background info on offender, and doing character assessment. These steps that will ultimately (informally) affect case outcome.
**Protected class**: First responders (police, fire, emergency medical service), teachers, doctors, and nurses.
**Victim's legal expectation**: Expectation of how the law should be held up in court based on what is told to the public.
**Victim investment:** How invested or concerned victim is regarding follow up of case and case details.
**Injury**: The circumstances of the physical injury. The severity of the psychological impact that injury has on individual.
**Offender‐focused approach/perspective**: Anything related to the justice system not made for the victim, but for offender rehabilitation and being a productive member of society.
**Offender characteristics**: Predictors/Factors about patient that may lead to different/special treatment due to underlying circumstances (mentally ill, homeless, medical disability, etc.)
**Community stress**: Perception of how stress levels may affect the community and things that may cause them to end up in the legal system.
**“Judge's effect”**: Bias or viewpoint held by judges that may change the final sentence of a case.
**Offender demographics**: Whether different factors like gender, race, or location alter when and/or how a case is prosecuted.
**Sympathy**: Acknowledgment of or lack of acknowledgment of the psychological impact an assault can have on the individual. Acknowledging that the severity of the injury impacts the charges/sentencing.

## 3 RESULTS

Themes identified in the analysis fell into two categories: factors that lead to a charge, and the judge's discretion in sentencing. The most salient themes that emerged from the first category were: prosecutorial merit, intent, and victim investment. In the second, a “part of the job” mentality, concern for the defendant, and the offender‐focused approach of the justice system emerged. Quotations included are identified using numeric values to preserve the anonymity of participants.

## 4 FACTORS THAT LEAD TO A CHARGE

### 4.1 Prosecutorial merit

DAs identified numerous factors that contributed to an assault not being charged as a felony. Themes such as the point at which things “fall out” of the prosecutorial process, whether intent can be determined, and the victim's investment in the process, all contribute to whether a charge is filed and the severity of the charge. For example, the amount of evidence to give a case “prosecutorial merit” is considered:

…a significant number of them fall out at the preliminary hearing for the following reason: that municipal court judges are charged by the law with determining one legal question. Is there sufficient evidence to prove a crime was committed and [that] that's the person [who] committed it? And it's by a preponderance of the evidence—more likely than not. What we see is **in cases where the crime is premised only on the protected status of the victim**… I don't know about the paramedics, but I do know that teachers and the police officers—**the municipal court judges don't follow the law the way it's written about protecting the people in the protected class**. (DA #1 [**emphasis** added])

And I've heard them make comments in individual cases that would lead you to believe that their perception is, well, when you take a job like that, you should expect to get hit. Ludicrous. Ludicrous. But municipal court judges in Philadelphia have had this tradition, some more than others, and some in more cases than others, of not only making a legal determination, but **they place a value on the case**, and they say **is this serious enough** to go to the Court of Common Pleas for a felony trial.” (DA #1 [**emphasis** added])

One DA described their discretion in interpreting whether an injury is serious enough to warrant a charge and that psychological injury to the victim is not considered at all or on a par with physical injury. The investigators discussed a previous paramedic assault case (prosecuted by the DA's office) in which a medic was hit in the eye socket resulting in a broken orbital bone:

DA #4: …it's all according to where you are in the criminal justice center. Most people would say that's not a serious injury. I sure would. Not that 30 stitches isn't a serious injury to most judges. Getting shot is a serious injury. Getting stabbed could be.

**Interviewer: How about a traumatic brain injury?**

DA #4: Really traumatic brain, what does traumatic mean? Does he just have some bleeding on the brain? Is he handicapped now for life?

**Interviewer: It has to be really bad.**

DA #4: Oh, yeah. Because they see really—if they're—we're all desensitized. So you have somebody coming in in a wheelchair, because they were shot, that's really bad. You have somebody coming in that was slashed on their arm with a knife, they got 30 stitches—is that so bad? I think it is. I would never want to deal with it. And most people that are getting slashed, this is their only time that anything like that has ever happened to them, but we see it every day. So it's kind of tough, trying to figure out what's—I thought a broken leg would be a serious injury. According to some people, that's not a serious injury.

**Interviewer: So the psychological impact of this experience to the medic is not even considered.**

DA #4: No.

**Interviewer: Or any first responder, doctor, nurse, police.**

DA #4: Nope. It might be considered at the sentencing if it comes to that. They're allowed to put the victim impact statement on. More times than not, that's a disadvantage for the medic or the police officer—for a first responder, because they're expected to be able to deal with that.

This issue of psychological injury arose in the interview with DA #4, and therefore, was revisited when interviewing DA #5:

DA #5: …but psychological, the issue with that, it's so hard to quantify. I can look at a broken bone or I can look at a contusion and know where I'm at. But for somebody to say, you know, it's really screwing with my head, how do you quantify, are you losing sleep? Or are you unable to function at your job? You know? It's hard to, I mean, maybe somebody is functioning in their job and still very upset by it.

### 4.2 Intent

Given that intent is a necessary requirement for a felony charge, DAs were prompted about the conditions necessary to determine intent.

Well, any crime requires a *mens rea*. I mean, any crime requires a guilty mind…and especially for assault—assault is a specific intent crime. You have to have the ability to form specific intent to cause bodily [injury] or serious bodily injury. So if we're missing that—we wouldn't be able to proceed at trial anyway. There's an *actus reus*—there's a guilty act, and then there's *mens rea*—there's guilty mind. So, if we don't have those two for any crime, how can we proceed?” (DA #2)

… in Pennsylvania to be charged with aggravated assault you have to find intent to cause serious bodily injury. Simple assault is intent to cause bodily injury. Simple assault is a misdemeanor. So to assault a paramedic, we don't need to prove that there was intent to cause **serious** bodily injury. We only need to show that there was intent to cause bodily injury. (DA #2 [**emphasis** added])

While felony assault to first responders requires only “bodily injury” intent, these interviews illuminated that findings of intent are rare due to the circumstances under which they often occur.

If you injure or attempt to injure somebody of the protected class, the law says you're guilty of felony in the second degree. What the fact finder—and that could be a judge or a jury—is enticed to believe by defense attorneys during trial is that it wasn't that big of a deal, and they didn't really intend to do it. And particularly with people who are being treated for injuries, is their action not a voluntary intentional act but in some ways an involuntary and/or an unintentional response to their trauma?

[Interviewer asks for an example]

DA#1: Overdose. The EMT administers Narcan. Person comes to. Ma'am, what's you name? I ain't telling you. Ma'am, I need to know your name. I need to know how you're feeling. I want to know your symptoms. Go fuck yourself. Ma'am, I understand you're upset. Boom. Smacked in the face to get up off the stretcher and leave.” (DA #1)

For medics and for firefighters, you know, the thing that kind of jumps out in my mind that we kind of see is, what was the person's mental state, you know? Were they really caught up in some sort of crisis where they might not have been thinking really clearly? Were they undergoing some sort of health issue where, you know, they're so worried about are they gonna die, the fact that they're flailing around hitting people is 95th on the list of things in their mind. So those are the kind of things we kind of try to look at. Did the incident happen right at the accident scene when they're trying to get this guy into the ambulance? Or was it 2 hours later at the hospital when the guy's, you know, kind of like, stabilized and now he's just being a jerk? (DA #5)

Certain conditions that might lead patients to become combative like mental illness, drug, or alcohol intoxication, and the underlying medical condition of the patient may interfere with the ability to form the requisite level of intent. Questions about establishing intent can cause cases to “fall out” of the prosecutorial pathway, leading to the emergence of nontrial disposition tracks.

These include diversionary solutions like Accelerated Rehabilitative Disposition (ARD), mental health court, drug, and alcohol rehab, and veterans court that are commonly used for restorative justice in lieu of proceeding to a felony charge.

### 4.3 Accelerated rehabilitative disposition

ARD is not a judicial—well, in the first instance, it's our decision. ARD is short for accelerated rehabilitative disposition. And it's a program designed for first‐time nonviolent offenders (DA #1).

From a perspective of ARD, what ARD is, is a statutory program that was, I think, maybe as far back as like 1972ish, was established by the legislature as, kind of like, the best description is a Get Out of Jail Free card. It's one of those ones where somebody had the foresight and common sense to say, you know, people have a bad day and some people have a really bad day, but it shouldn't haunt them for the rest of their lives. So it's a mechanism by which a person can be held accountable, through the courts, for whatever action they were arrested for, not have a conviction result in that act, but also be held accountable, generally through some sort of probation requirements (DA #5).

### 4.4 Drugs and alcohol

Again, if they're under the influence of drugs and alcohol, I think that turns into a situation where, you know, how harmful was their action, you know? Was it a push? Was it a punch? Was it a kick to the face? Was it a kick to the groin for a guy? Or was it just a general flat lashing out? You try to look to see if there's any intent. And it's hard. **When somebody's on drugs, you know, do they have intent?** (DA #5 [**emphasis** added])

…for the judge, the main issue was that he was so intoxicated that she said that the **defendant would not have known** that he was assaulting someone who is a protected class—which I don't think is a standard, and so I disagree with that. But that's the verdict that we were left with. (DA #3 [**emphasis** added])

Unless the violence involves a weapon or premeditation, intent is not going to be established, and therefore a felony charge cannot proceed. This quote from one of the participating DAs summarizes the difficulty prosecuting and convicting cases and expresses how difficult that can be for victims to accept or understand.

… I don't think any civilian victim should or perhaps can have this perception that if they are the victim of a crime that automatically their perpetrator will be found, identified, prosecuted and convicted—and sentenced to jail time. For all of those steps to happen, that's—it's hard. It's hard to do that. And does it happen in a majority of the cases? I don't know. I don't know what the numbers are. I think the paramedics should know that someone is always looking at these cases, reviewing these cases, taking them seriously, handling them the best way they see fit. But as far as the outcome goes, that's really not in the hands, certainly, of the district attorney's office. And I think they know that. It's a much wider and larger systems issue than how the case—how the case is handled, perhaps, is different from the outcome of the case, unfortunately. (DA #2)

### 4.5 Victim investment (responder involvement)

After an assault‐related injury, it can be very difficult for first responders to seek justice due to resulting physical and emotional pain, the financial loss of taking leave from work, or a loss of faith that the system will actually give them the results they expect. Any perceived lack of investment on the part of the first responder may result in a lesser charge being offered. This is simply a reflection of the pipeline in the DA's office—there are so many cases to charge and process. If the victim does not press charges and stay involved, the prosecutors may feel compelled to move on to the remainder of their workload.

…and I don't know that the department has sort of a no‐tolerance policy that if a member of the department is assaulted that they have to report it to Philadelphia police. I'm guessing that even if they do, that's not strictly enforced. So there's some discretion on the part of the person who's assaulted whether they want to involve the police. (DA #1)

DA participants indicated that throughout the lifespan of a case, the amount of dedication and investment that the victim has in the details of the case can alter its outcome:

I wonder if we have any issues with the paramedics coming to court…because we're either taking them away from work, if they're otherwise scheduled to work, or we're taking them from their free time, which is precious and scarce as it is. And if they get to the point, well, you know what? It wasn't really that big a deal. I don't care what they do with this guy, but I'm not going. And in fairness to them and every other victim, **the criminal justice system is not victim friendly.** You come into a courtroom. You've got to be there at 9:00. You have no idea when your case is going to be heard. You sit in the audience where your offender could be sitting in the row in front of you or behind you. We can't tell you how long you're going to be there or what order the cases are called. And you go in and you're asked a bunch of questions. And sometimes you're treated appropriately, sometimes you're not, and then the judge makes the decision. So if you go through this once where your case gets thrown out, I could understand why they would get dissuaded and say screw it, I'm not going. (DA #1 [**emphasis** added])

If you have 50 cases on the list and you have a guy—a victim who's like I can't come back after this, I've taken so many days off. You might offer something a little bit lower than you normally would—if the person pleads guilty. Mostly because you know the defendant did it, but if a case gets continued, and this person's not coming back, then this case is gonna be disposed. (DA #3)

Getting anyone to come to court is hard. Getting police officers to come to court is hard. Getting first responders to come to court is hard. They think that—as far as I know, they think it's more important for them to be at their job than wasting all day in court which I can't blame them, but that's why we give probation offers, because they don't come to court. And I don't mind giving a probationary offer if they're not coming to court. Because I'm getting the guilty and I'm getting this guy supervised for the next 4 years. If they want more attention paid to their case, they either have the supervisor call and they have to contact the DA themselves. They have to show some interest. When a civilian shows an interest in a case, we really pay attention to that case. If an officer shows attention to a case—a shooting, especially—we pay attention to the case. If it's a simple assault with a civilian, and they call, “oh, do I really have to come?”…What are we going to do? Why should we care anymore than them? (DA #4)

### 4.6 Preparation for court

Despite the importance of the victim's participation in the judicial process, the analysis identified a perception that first‐responder victims should be sufficiently familiar with the process to deal with it without instruction or preparation.

…I would hope that they're getting justice the way it is. I mean, if you're going to trial—it's the perception on the sentencing part by the judge that you should be expected to be able to deal with this. That might be why the sentence is lower. It's the perception by our DAs that I don't need to prep you—or even talk to you, because…you should know already. (DA #3)

You expect them to have the testify in court down, because they're in uniform. Because they're a city employee. And it's not the way it is. It's just not. That's why a prep is so important with them. And it's hard to sit down and prep, because you have 2 minutes in the back room and the judge is screaming for you. It's really frustrating. It is. It's—when you're over there, nothing is about the victim. **Nothing is about the victim**…It's about how I'm going to get that guy…it's all about the defendant. (DA #4 [**emphasis** added])

But sort of the presumption is if you're a professional like a paramedic, well, you're not going to need handholding because you're different than Susie Grandmom whose house got burglarized. She's 78 years old. She's going to need some handholding. Or a 14‐year‐old girl who's sexually assaulted or a guy who's beaten over the head while he's on a subway and his wallet's taken from him. So we're doing 50 000 criminal cases a year in Philadelphia. Just like you can't run a hospital and treat everybody like a heart attack patient, because they're not. I'm sorry. (DA #1)

Preparation for court, coupled with strong victim investment, can affect the prosecutorial efforts to get the sentence that is most satisfying to the victim.

## 5 JUDGE's DISCRETION IN SENTENCING

In addition to the factors that can determine whether or not a charge is filed, there are factors that can influence whether a felony sentence is served. The data identified that a judge's discretion includes a “part of the job” mentality, concern for the defendant, and the fact that the justice system has an offender‐focused approach. These all play a role in determining how a judge decides to sentence a case.

### 5.1 “Part of the job” mentality

District Attorneys described a particular viewpoint held by some judges: that violence against first responders is “part of the job.” This viewpoint was offered as explanation for why violent acts against first responders do not result in felony convictions despite the victims' protected status.

“**That's their job.**” Hear that all the time. Especially with police officers that are punched, that are cut, something. Judges all the time say, ‘that's what he signed up for. That's his job. This shouldn't be a felony. Why is it a felony? He knew what he was getting into.' I used to say to judges—‘judge, if I jumped over the bar of this court and punched you in the face, I would hope that that would be a felony. That's what you're saying isn't a felony.' And I've had judges, ‘oh, that's a good argument. No, denied. Yup.' (DA #4 [**emphasis** added])

So, do I think that there's a sentiment among, perhaps, the judges that says, you know, guys, whether it be police, fire, paramedics, even teachers to an extent, **you signed up for this**? You're in a big, ugly city. You're in a big, violent city. If you're dealing with people who are high or drunk or have these problems, it's almost an assumption of the risk. It's almost, you know what you're getting into when you sign up for this job. Thank God you weren't seriously hurt. Thank God you didn't miss any time from work. Okay. We're here acknowledging you. Now let's move on and get to the person whose house was burglarized and they haven't been able to sleep there for 2 weeks. (DA #2 [**emphasis** added])

These data highlight that while first responders may be a protected class under the law, judges still bring their own perspectives when determining the sentence for each case and often times believe that under the circumstances, violence is to be expected.

### 5.2 Concern for the defendant

DAs explained how the gravity of a felony sentence, and its subsequent detrimental effects on the perpetrator's life, can influence how a judge determines sentencing of these cases. Because judges understand how debilitating a felony sentence can be for an individual, they have inherent concern for the defendant. Given that some judges also may feel as though violence is “part of the job” for a first responder, concern for the victim is often not reflected in their decisions.

…(I'm treating a patient and I get) smacked in the face (when they try) to get up off the stretcher and leave. The law says that's a felonious assault. A lay person may say, well, that ain't right but it ain't a felony either. Not just the lay person on the jury, but more commonly a judge, **because the lay people in a jury don't know the consequences that the defendant's going to face if convicted. A judge does**. (DA #1[**emphasis** added])

What are the judges thinking? Where is this case gonna go from the judges? My history in the court system in Philadelphia is that if you take this case to trial, the majority of these cases are not gonna get held for court as felonies and then those that do get held for court as felonies are, even if they're found guilty… they're not gonna be found guilty as a felony aggravated assault…I can guess that the judges are probably of the same mindset, saying this person was probably, you know, didn't have the intent to really hurt someone where and, you know, I'm gonna split the difference, you know? I'm gonna hold him accountable, give justice to the first responders, but at the same time, not really jam the guy up. I think there's some mindset of officials on the bench, and also our office, too, quite frankly, me, I don't want to put a felony conviction on somebody that doesn't really deserve a felony conviction. **A felony conviction is like a death sentence to somebody's life**, in some respects. If you have a felony conviction, you're never getting a good job, straight up, they're gonna run a background check and see you have a felony conviction for aggravated assault, who's hiring you? So now that person doesn't have a job. So now that person's on state support and his family's on state support. And who pays for that in the end? The taxpayers do. We all do. **So is it really worth jamming this guy up with a felony conviction over one bad incident?** (DA #5 [**emphasis** added])

These quotes express the severity of a felony conviction in that it can change the course of a person's life. It can prevent a person from getting a job and cause them to be unproductive in the community, so those in the criminal justice system try not to deliver that punishment unless absolutely necessary.

### 5.3 Offender‐focused approach

Data from the Philadelphia DAs office revealed that its criminal justice system is not designed for the victim, but rather it has an offender‐focused approach. Its system is more focused on the rehabilitation of the defendant to ensure that whatever brought them into the criminal justice system will not occur again in the future, rather than to serve justice and make the victim feel whole again. This system can often be dissatisfying to the victim, with one DA describing the experience for them as: “The crime has been devalued, and as result, they feel devalued” (DA #1). This same DA explained that most victims, first responders included, are not pleased with the results of their case through the criminal justice system, “I'd be shocked if we found any significant number of victims, paramedics or otherwise, who are pleased with their treatment by the criminal justice system, because it's not—it's fundamental. **It's not victim centric**” (DA #1 [**emphasis** added]). Indeed, the first time a person is processed by the criminal justice system (eg, first offense), there is a strong proclivity to not have that result in a conviction if the person appears to be reachable through rehabilitation:

What we try to do in every case are three things—at least three things. First is protect the community. Second is hold the offender accountable to the community and to the victim. And third is help the offender become a responsible productive citizen. If you take the same philosophy that articulated in charging, not every violation needs to be—needs to result in an arrest and prosecution. Not every arrest needs to result in a conviction. So, based on what we know about the offender and what we know about the offense, we say to the offender's lawyer, look, if he agrees to be on probation for a set period of time up to 2 years, and do a number of things; not break the law, not get arrested, if necessary drug and alcohol counseling, mental health counseling, stay away from the victim, pay restitution, all those sorts of things for the probation period, at the end of the probation period, we will withdraw the charges. So it's a second chance for a first offender. (DA #1)

## 6 DISCUSSION AND RECOMMENDATIONS

The state of Pennsylvania expressed its values by making first responders members of a “protected class,” elevating assaults against their person from simple to aggravated and thus felony status.^12^ While it is important to have felonious assault statutes as an expression of society's value that it is unacceptable to harm a first responder, the realization that many cases do not result in felony sentences removes the hope of procedural justice from first responders.

The criminal law serves multiple functions. It is intended to punish the wrongdoer but in so doing, it is also intended to deter others from wrongdoing. Studies of the deterrent function of criminal law have suggested that its effectiveness rests on the perceived likelihood of getting caught rather than on the severity of the statutory punishment.^13^ This suggests that to the extent that felonious assault statutes inadvertently decrease the likelihood of conviction in exchange for increasing the severity of punishment, they will fail to enhance deterrence. But the criminal law often serves an expressive function as well. “Rather than the particular consequences of an action, the concern instead is with upholding certain values and norms through the law, even if the consequences of the passing of such laws are unknown and obscure”.^14^ In this view, even if the felonious assault laws fail to achieve a deterrent effect on violence against first responders, they may still serve a function of articulating the value of the importance of first responders to society. However, this function may be undermined when the law is not enforced. A lack of enforcement—or weak enforcement—may communicate to first responders the reverse message: that they are not valued enough for the protection they were promised.^15^

**Figure 1:**
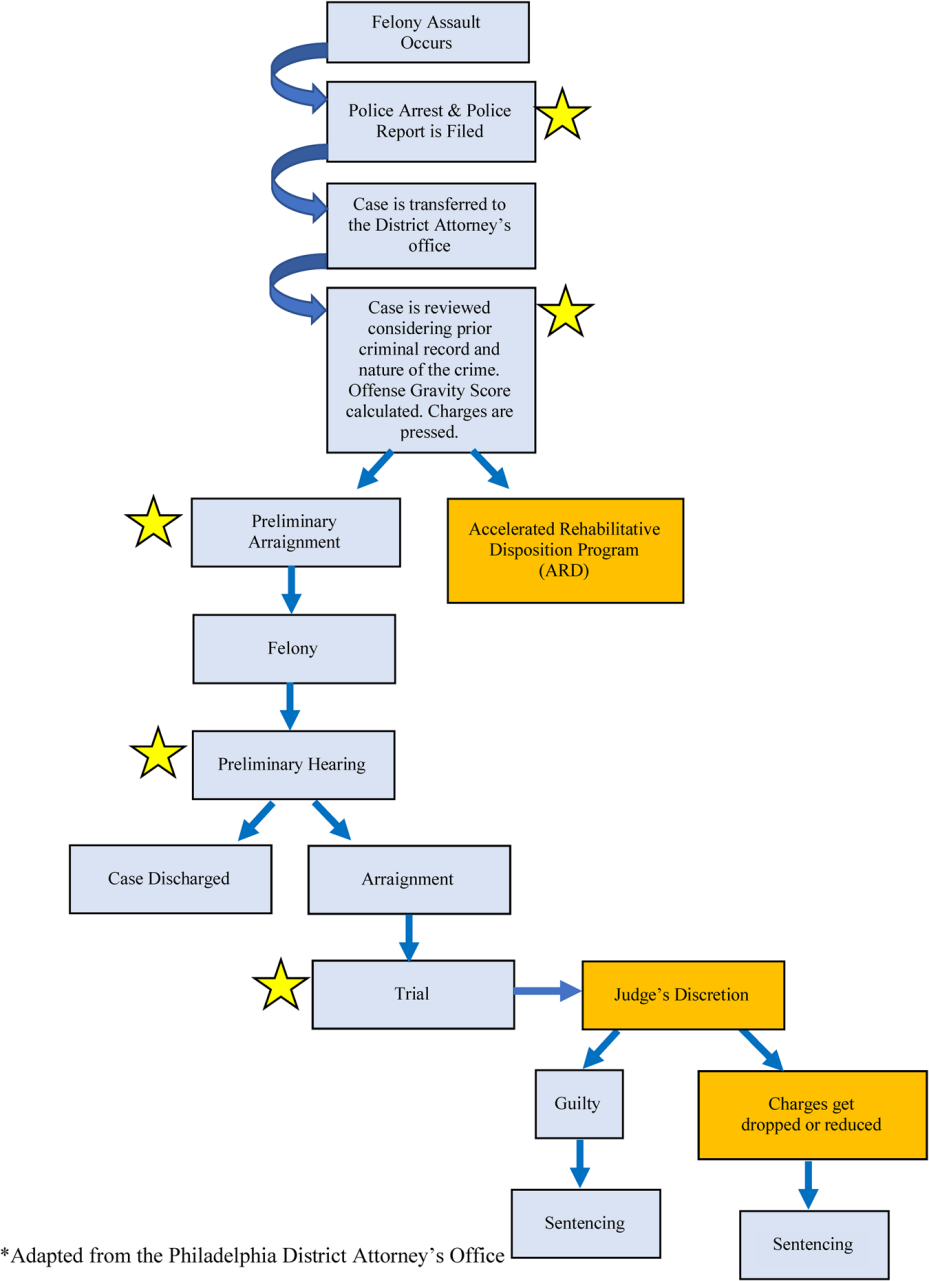
The prosecutorial pathway [Color figure can be viewed at wileyonlinelibrary.com]

Figure 1 illustrates the prosecutorial pathway that a case would take when a first responder is assaulted while on duty. The blue portions of the flowchart are the law as currently written. These are also the steps that first responders expect to happen, given their protected class status and understanding that assaulting a protected class is a felony. However, our research clarified that the orange portions of the flowchart are also very much in play and can greatly influence the decision as to whether a case is tried and sentenced as a felony or not. At the time that a case is reviewed by the DA's office, many different factors are considered which can change the final outcome, including a person's criminal background, character assessment, nature of the crime, and the discretion of the judge. At this step, the DA can offer the defendant ARD rather than moving to a preliminary arraignment. Another point where a case can “fall out” of the prosecutorial pathway is at trial when judges enact their discretion over the case. At this time, charges can be dropped or greatly reduced. These orange‐highlighted decision points deviate from the expectations first responders have, given their protected status. Our research illustrated that these areas of the prosecutorial pathway need to be clearly described to first responders so that they have realistic expectations of the judicial system and the outcomes that their cases may receive.

Yellow stars have been added to points in the pathway where organizational support and responder involvement are critical to achieve the desired outcomes. In order for any of these steps to be set in motion, a police report must be filed by the injured first responder. As we learned from our participants, the first responder also needs to be invested in the process at the time that the case is reviewed to ensure that the prosecutor fully understands the severity of the experience for the responder and their desire for full prosecution. All of the starred steps must have strong organizational support extended to the first responder so that they feel that they are not navigating the prosecutorial landscape in solitude.

In the interviews with Philadelphia District Attorneys, findings of intent were found to be rare because of the context in which these cases arise. This removes the ability of assaults against first responders to be prosecuted as felonies. Many of the perpetrators had medical issues (diabetic shock and dementia), mental health issues, and drug or alcohol conditions that prevented an “intent” determination. Because of these reasons, cases “fall out” of the prosecutorial pathway, and nontrial disposition tracks emerge. These include alternative processes that address and rehabilitate the offender, and take the forms of ARD, vet court, mental health court, and drug/alcohol rehabilitation. First responders previously reported their dissatisfaction with an ARD solution.^1^

The current investigation found that there are many factors that lead to a charge such as the strength of evidence, the severity of the injury to the first responder, if intent can be determined, and the first responder's investment in the process. It also identified that judges may believe that violence is an expected part of a first responder's job, that judges may have more concern for the perpetrator than the victim, and that the offender‐focused nature of the legal system influences sentencing in an unsatisfactory way for the victim. As expressed by one of our participants, “Only 1% of criminal cases go to a jury” (DA #1).

An important limitation of this study was that only DAs were interviewed. The perceptions they shared on how judges act and think are theirs alone. Furthermore, time, resources, and DA workload limited our inquiry to snowball sampling five out of approximately 300 DAs.^16^ Snowball sampling was particularly useful in this study design given that the interviewees needed to have experience with cases involving first responders and this sampling technique yields individuals who will be expertly able to speak to the research question.^17^ While this was an exploratory study that never intended to randomly select from all DAs, we were satisfied with the diversity of interviewees because they represented distinct and important perspectives in the prosecutorial process such as those that are considered as cases make their way through the trial, charging, and diversion divisions. A senior DA from the administration division made the recommendations of whom we should contact given our goals to understand the prosecutorial process and we trust that direction since it aligned with divisions mentioned in the published pathway (Figure 1). The authors also felt no additional interviews were needed from DAs since data saturation based on our interview guide was achieved: by the fifth interview, no new themes emerged.^18^ One municipal court judge was interviewed, but since we were unable to secure other judges' participation, we did not feel we had sufficient data to include that perspective in this manuscript.

There are other limitations to consider. This was an exploratory qualitative study. It describes only what happened with treatment of felony offenses to EMS responders in Philadelphia and is not necessarily generalizable to the state of Pennsylvania or any other state, metropolitan area, or rural community. Future research should address the representativeness and generalizability of the present study's findings to understand how the prosecutorial process works in different states, and in jurisdictions with smaller caseloads, and lower incarceration rates. As we were focused on firefighters and EMS responders alone, we do not know if there are differences in how the felonious process works for other commonly assaulted first responders including police, doctors, nurses, or teachers. What is clear is that these types of statutes exist in many states across the country.

So, what can be done to address the frustration and dissatisfaction of assaulted first responders? While the best solution would be to prevent these assaults from happening in the first place, the present study focuses on problems present with current tertiary prevention solutions. Tertiary prevention tries to stop present damage from worsening and emphasizes recovery and rehabilitation from harm. We present three recommendations for fire departments, labor unions, and the criminal justice system to consider.

### 6.1 Recommendation #1: Educate DAs, judges, and defense attorneys that violence to first responders is not “part of the job” by communicating the impact of violence

Reflecting on the sentencing part of the process, District Attorneys shared numerous instances of their belief that they and judges have internalized violence to first responders as part of the job: “When you take a job like that, you should expect to get hit” (DA #1), “That's what he signed up for. That's his job” (DA #4). This is a concerning finding, yet not surprising given the volume of cases the district attorney and municipal court system must process. The investigators observed that this intense caseload and job demand leaves them with little excess time to analyze cases. Participants exhibited a level of desensitization to the severity of the physical injury (“Oh, yeah…we're all desensitized”—DA #4), and really no awareness of the psychological injury that so often accompanies a physical one (“they're expected to be able to deal with that”—DA #4). The current Pennsylvania statute is silent on this “second injury.” Even though first responders are often working in “big, ugly cities,” violence on the job is not an acceptable attribute of their work environment.

### 6.2 Recommendation #2: Develop education and training to prepare assaulted EMS responders for court appearances through communication and collaboration among the DA's office, fire department management, and the labor union

This idea of a second injury can be extended from the assault to the time when a first responder goes to court alone and unprepared for what is an unfamiliar proceeding. The DAs recognized this, “You sit in the audience where your offender could be sitting in the row in front of you or behind you” (DA #1). While the DAs thought first responders should be ready for court appearances (“You're not going to need handholding”—DA #1), they do not have training on navigating court proceedings like other members of the protected class (eg, police, fire marshal). Therefore, support for court is needed, including preparation for court appearances and attendance by members of the fire department and labor union to support the assaulted member during the proceedings. This can take the form of a standard operating procedure such as that released by the Philadelphia Fire Department in July 2018 (after the data presented in this manuscript was collected) that expresses the organizational commitment to the safety of its membership and lays out a protocol for how assaulted responders will be supported by fire department and union leadership (PFD Operational Procedure #42—Procedure for Member Assaulted on Duty [2018], Fire Commissioner Adam Thiel, personal communication, 27 March 2019).

An inspiring moment came when one of the DAs said they would be happy to visit with members and prepare them for court. “I think it's helpful just for me to have a conversation with them, because they don't know how the system works. So that they can have a decent handle on what goes on in the criminal justice system” (DA #1). This DA went on to say that they would be interested in meeting with them to help them learn the process to “dispel the myth of the unknown.” But as identified by the DAs, the victim needs to stay invested throughout the process in order for the charges to be satisfactory to the victim.

### 6.3 Recommendation #3: Develop nonpunitive leave policies that support the injured EMS responder

In addition to the physical and psychological burdens of experiencing a work‐related assault, there can be financial burdens for EMS responders. Victims may have to take unpaid time from work for multiple court appearances, may not have “stress pay/mental health days” as part of their benefits system, and may not work in environments that have expressed statements about “zero tolerance” for violence against employees. Such policies would likely help keep the victim invested in the process. While they may not change the result in court, they may prevent first‐responder victims from feeling entirely abandoned, thus serving the goal of supporting them in dealing with the stresses of the assault, the legal process, and challenging working conditions.

## 7 CONCLUSION

In this study, a tension was found between society's desire to turn the perpetrators of crimes back into productive citizens and providing justice to the victim. Victims feel underrecognized and abandoned. Perpetrators want a second chance. District attorneys and judges are saddled with an overwhelming caseload and the desire not to burden a perpetrator with a felony sentence that will remove from them the opportunity for rehabilitation. This taxing caseload may lead to detachment that may affect interpretation about the severity of an injury and the intent of the perpetrator.

The recommendations provided herein would improve the way the system serves injured first responders. But at the end of the day, the criminal law system is likely to be an unsatisfying one for assaulted first responders. First responders should be given more satisfying solutions. Therefore, the focus should shift from passing more felonious assault and other criminal statues, to primary prevention interventions that can help prevent violent encounters from occurring, precluding first responders from having to seek satisfaction from the prosecutorial process in the first place.

*This article is dedicated to the memory of its first author, Jasmine Yolanda Wright, MPH*

**In Memoriam: Jasmine Y. Wright, MPH ∼11.23.1987 – 07.16.2015**

Jasmine Yolanda Wright, MPH was the first author of this manuscript. These results are the findings of her Master s in Public Health thesis in Environmental and Occupational Health, conducted at the Dornsife School of Public Health at Drexel University. This paper explains that in Pennsylvania it is a felony to assault a first responder. But when we interviewed paramedics, we heard that that prosecutorial process actually made them feel injured all over again. Jasmine set out to find out why. She did that by looking at the transcripts of what the paramedics said, and then by interviewing district attorneys in Philadelphia. Now understanding a district attorney is hard enough because it's like they speak a different language but getting a district attorney to talk is actually the hardest part. This is what was really important about Jasmine. She never gave up. She would call, she would email, she would show up. She would get these people to talk to her. And they could see her passion and they shared with her the intricacies of their discipline and the limitations on what they could do to bring justice to victims.

One month after her MPH graduation in 2015, Jasmine was murdered. Our team was too aggrieved to return to this work. But as the fire service needs to understand these results, and it was the best way to honor her memory, we endeavored to revise the manuscript. Most of her original thoughts and writings are here, but because it had been years, we went back to the source data and made additional observations, incorporating those with her findings.

Jasmine was committed to the US fire service, to injury prevention, and to occupational safety and health. We all know the personal joy we take when one of our students chooses our disciplinary path. She also wanted to fight for justice. She chose to stand for the paramedics who are called to our homes not knowing what they are about to walk into. The experience she had working with the Philadelphia District Attorney s Office inspired her plan to pursue a law degree so that she could provide critical policy analysis on occupational hazards like assaults. This is the year she would have graduated.

The other thing Jasmine's research showed us, is that in those rare cases where a patient intended to hurt the paramedic caring for them, a felony conviction can be attained only under certain conditions. Jasmine's research showed that the victim and their families needed to stay close to the prosecuting attorneys to ensure that the case received the attention and the sentence that they felt gave them justice from their injury. But primarily, what Jasmine learned about the law and its punishments is that it is only in prevention that we are satisfied. Jasmine learned that paramedics assaulted by patients will really only ever be well if the injury never happens in the first place.

We remain overwhelmed by the irony that Jasmine was declared ‘deceased’ by the very people she committed her public health career to protect.

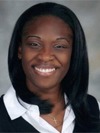

## CONFLICTS OF INTEREST

The authors declare that there are no conflicts of interest.

## DISCLOSURE BY AJIM EDITOR OF RECORD

Paul Landsbergis declares that he has no conflict of interest in the review and publication decision regarding this article.

## AUTHOR CONTRIBUTIONS

JYW and JAT conceptualized and designed the research question which served as JYW's Master's Thesis work at the Dornsife School of Public Health. JYW, JAT, and ALD contributed to the acquisition, analysis, and interpretation of the qualitative data. All authors contributed to framing and writing the manuscript. JYW drafted the early versions of this manuscript and then JAT, ALD, and SBR revised and added to the manuscript to achieve this submitted version. JAT, ALD, and SBR have given final approval of this version to be published and they agree to be accountable for all aspects of the work and will answer any questions related to the accuracy and integrity of the data presented in this manuscript.

## ETHICS APPROVAL AND INFORMED CONSENT

The Drexel University Institutional Review Board approved this study. Written informed consent was obtained from each participant.
